# Validation of the Prediction Accuracy for 13 Traits in Chinese Simmental Beef Cattle Using a Preselected Low-Density SNP Panel

**DOI:** 10.3390/ani11071890

**Published:** 2021-06-25

**Authors:** Ling Xu, Qunhao Niu, Yan Chen, Zezhao Wang, Lei Xu, Hongwei Li, Lingyang Xu, Xue Gao, Lupei Zhang, Huijiang Gao, Wentao Cai, Bo Zhu, Junya Li

**Affiliations:** Laboratory of Molecular Biology and Bovine Breeding, Institute of Animal Sciences, Chinese Academy of Agricultural Sciences, Beijing 100193, China; jiujiuyake@sina.com (L.X.); nqh_5195@163.com (Q.N.); chenyan0204@163.com (Y.C.); wangzezhao1@163.com (Z.W.); xuleirock@163.com (L.X.); lihongweicaas@163.com (H.L.); xulingyang@163.com (L.X.); gaoxue76@126.com (X.G.); zhanglupei@caas.cn (L.Z.); gaohj111@sina.com (H.G.); caiwentao@caas.cn (W.C.)

**Keywords:** genomic prediction, prediction accuracy, low-density SNP panel, Chinese Simmental beef cattle

## Abstract

**Simple Summary:**

To reduce the breeding costs and promote the application of genomic selection (GS) in Chinese Simmental beef cattle, we developed a customized low-density single-nucleotide polymorphism (SNP) panel consisting of 30,684 SNPs. When comparing the predictive performance of the low-density SNP panel to that of the BovineHD Beadchip for 13 traits, we found that this ~30 K panel achieved moderate to high prediction accuracies for most traits, while reducing the prediction accuracies of six traits by 0.04–0.09 and decreasing the prediction accuracy of one trait by 0.2. For the remaining six traits, the usage of the low-density SNP panel was associated with a slight increase in prediction accuracy. Our studies suggested that the low-density SNP panel (~30 K) is a feasible and promising tool for cost-effective genomic prediction in Chinese Simmental beef cattle, which may provide breeding organizations with a cheaper option and greater returns on investment.

**Abstract:**

Chinese Simmental beef cattle play a key role in the Chinese beef industry due to their great adaptability and marketability. To achieve efficient genetic gain at a low breeding cost, it is crucial to develop a customized cost-effective low-density SNP panel for this cattle population. Thirteen growth, carcass, and meat quality traits and a BovineHD Beadchip genotyping of 1346 individuals were used to select trait-associated variants and variants contributing to great genetic variance. In addition, highly informative SNPs with high MAF in each 500 kb sliding window and in each genic region were also included separately. A low-density SNP panel consisting of 30,684 SNPs was developed, with an imputation accuracy of 97.4% when imputed to the 770 K level. Among 13 traits, the average prediction accuracy levels evaluated by genomic best linear unbiased prediction (GBLUP) and BayesA/B/Cπ were 0.22–0.47 and 0.18–0.60 for the ~30 K array and BovineHD Beadchip, respectively. Generally, the predictive performance of the ~30 K array was trait-dependent, with reduced prediction accuracies for seven traits. While differences in terms of prediction accuracy were observed among the 13 traits, the low-density SNP panel achieved moderate to high accuracies for most of the traits and even improved the accuracies for some traits.

## 1. Introduction

Genomic prediction (GP), which uses genome-wide markers to predict direct genomic estimated breeding values (DGVs), has been widely studied in breeding programs for plants [1,2,3] and domestic animals [4,5,6]. Recently, advances in high-throughput genotyping technology and the availability of population-scale whole-genome sequencing (WGS) have contributed to the identification of millions of genetic variants. Theoretically, the accuracy of GP depends on the degree of linkage disequilibrium (LD) between markers and quantitative trait loci (QTLs). In real-world contexts, the true QTLs are unknown, and markers must be selected as proxies to explain genetic variance [7,8]. In this setting, increasing the available markers could allow all QTLs in LD to be linked with at least one marker, which in turn would be beneficial for the GP. The application of high-density SNP arrays (770 K) for GP in cattle has been gaining increasing popularity because the high number of markers may improve the prediction accuracy of DGVs [9,10,11,12]. Previous studies have demonstrated that the usage of high-density markers (~770 K) can contribute to an improvement of prediction accuracy in comparison with moderate- (~50 K) or low-density (~30 K) markers [5,9,13,14]. However, this increase is limited compared with the high cost of genotyping. In fact, a moderate marker density (like 50 K) is enough for routine genomic predictions and can achieve satisfactory accuracy [15,16]. The BovineSNP50 Beadchip is one of the most popular SNP arrays used in the genomic prediction of dairy cattle [17,18]. Calus et al. (2008) demonstrated that 30,000 markers were sufficient to obtain accurate DGVs in a Holstein–Friesian cattle population where the mean LD (*r*^2^) between adjacent SNPs was 0.2 at approximately 100 kb. Vazquez et al. (2010) found that using 10,000 markers resulted in great prediction ability for production traits in Holstein cattle, and that the prediction ability was not improved as the number of markers continued to increase [19].

Many studies have been performed to develop a low-density SNP chip for cost-effective prediction in cattle [20,21,22,23], sheep [24], and chicken [25]. These studies can be roughly divided into two categories. One is to develop a low-density SNP chip by the selection of SNPs with nearly equal spacing, high minor allele frequency (MAF), or above a LD threshold, followed by the imputation of the low-density genotype to high-density genotype for GP [20]. The performance of these low-density SNP chips heavily relies on the imputation accuracy. Nevertheless, the imputation accuracy is influenced by many factors, including size of reference population for imputation, minor allele frequency (MAF) of the imputed SNPs, LD, and the relationship between reference and candidate population. Another strategy is to develop a trait-specific low-density panel by selecting a subset of SNPs on the basis of whole-genome regression analysis [21,22,23]. However, the number of preselected SNPs is small, ranging from tens to hundreds, since only one or two traits are used for the panel’s development. The loss of prediction accuracy would be large when using it for GP. In addition, the trait-specific panel could only be applied for a limited number of traits, unless the preselected SNPs for various traits are integrated to form a comprehensive low-density SNP chip. For these studies, the main hurdle for the application of these low-density SNP chips in GP is that they only focus on one purpose for low-density SNP chip development, i.e., either imputation-based or trait-specific cost-effective GP.

In China, the efficient genetic improvements brought about by GP and trends related to decreasing genotyping costs in contrast to increasing expenses for phenotyping have encouraged the application of GP to beef cattle. However, the cost of genotyping is still very high for breeding organizations in China; for instance, the cost of genotyping of BovineHD Beadchip is around 150 USD per animal. However, the cost of the low-density SNP chip is only 30 USD per sample. Chinese Simmental beef cattle play a dominant role in the Chinese beef industry because of their high adaptability and marketability. Simmental cattle and their crossbreeds account for more than 60% of the total cattle breeding stock across the country [26]. The primary breeding objective of Chinese Simmental beef cattle is to improve beef production and meat quality. The Genomic China Beef Index, which contains the official selection criteria for beef cattle breeding, consists of several genomic estimated genomic breeding values associated with economical traits, including calving difficulty, weaning weight, average daily gain during fattening, carcass weight, and dressing percentage. To reduce the breeding costs and promote the application of GP in Chinese Simmental beef cattle, it is necessary to develop a special low-density SNP panel which can achieve satisfactory prediction accuracy. Breeding organizations would then have access to the low-density SNP panel, which would provide them with a cheaper option with greater returns on investment. In this study, 13 growth, carcass, and meat quality traits and BovineHD Beadchip genotyping of 1346 individuals were combined to develop a customized low-density SNP panel for Chinese Simmental beef cattle via four variant selection strategies. Overall, the objectives of this study were to achieve accurate prediction of DGVs for economic traits of Chinese Simmental beef cattle by using the customized low-density SNP panel (~30 K) and to reduce the breeding costs by promoting the cost-effective GP.

## 2. Materials and Methods

### 2.1. Ethics Statement

All the animals used in the study were treated in accordance with the guidelines established by the Council of China Animal Welfare. The protocols of the experiments were approved by the Science Research Department of the Institute of Animal Sciences, Chinese Academy of Agricultural Sciences (CAAS) (Beijing, China). The approval ID and permit numbers are SYXK (Beijing) 2008-007 and SYXK (Beijing) 2008-008, respectively.

### 2.2. Animals and Phenotypic Data

A total of 1346 Simmental cattle born between 2008 and 2015 were collected. Of the sample, 1114 animals were bulls. All individuals were collected from farms in Ulgai, Xilingol League, and Inner Mongolia of China, and they were transferred to Beijing Jinweifuren Farm for fattening under the same feeding conditions and measured for growth traits every 3 months. Then, a slaughter experiment was performed for various phenotype measurements when the cattle reached an average age of 20 months. A more detailed description of the management processes was reported in previous studies [27,28]. This study analyzed the following 13 traits: (1) growth traits: average daily gain (ADG; kg), live weight (LW; kg); (2) carcass traits: hot carcass weight (CW; kg), dressing percentage (DP; %), lean meat percentage (LMP; %), and weight of retail beef cuts, including striploin (ST; kg), spencer roll (SR; kg), chuck roll (CR; kg), and tenderloin (TD; kg), and retail meat weight (RMW); (3) meat quality: eye muscle area at the 12th rib (EMA12, cm^2^), eye muscle area at the 13th rib (EMA13, cm^2^), and marbling (MB). The ADG was the rate of weight gain per day over the fattening duration. LW was measured before slaughter after fasting for 24 h. MB was visually scored on a seven-point scale depending on the degree of marbling on the cut surface of the 12th rib. In terms of the rib eye area (REA), a piece of the meat about 3 cm thick between the 12th and 13th rib was cut from the carcass. The rib eye area of both sides of the meat was measured as the area in square inches using a grid like the one pictured, and the two measurements were termed EMA12 and EMA13, respectively. Carcass traits and meat quality traits were measured in strict accordance with the guidelines proposed by the Institutional of Meat Purchase Specifications and GB/T 27643-2011 after slaughter.

### 2.3. Genotyping and Population Structure

DNA was extracted from blood samples via routine procedures and genotyped using the Illumina BovineHD Genotyping Beadchip. Raw genotype data were processed using PLINK (v1.90) before statistical analysis [29,30]. Individuals and autosomal SNPs that failed in any of the following criteria were discarded, resulting in 1331 individuals and 671,204 SNPs left: SNPs call rate >0.90; minor allele frequency (MAF) >0.01; *p*-value of Hardy–Weinberg equilibrium (HWE) chi-squared test >10^−6^; individual call rate >0.90. Alongside the quality control, missing genotypes were imputed using BEAGLE v4.1 software [31]. The SNP configuration was coded as the number of copies of the minor alleles, i.e., 0, 1, and 2 for the first homozygote, the heterozygote, and the second homozygote, respectively. To capture the population structure, we performed the principal component analysis (PCA) and linkage disequilibrium (LD) analysis using the PLINK (v1.90).

### 2.4. Genetic Parameter Estimation

The genetic heritability was estimated via the single-trait animal model in ASREML (v4.1). The genetic correlations and phenotypic correlations of 13 traits were estimated using the bivariate animal model in ASREML (v4.1) [32]. The bivariate animal model used for genetic correlation is described below.

(1) $$\left[\begin{array}{c} y_{1}\\ y_{2} \end{array}\right]=\left[\begin{array}{cc} X_{1} & 0\\ 0 & X_{2} \end{array}\right]\left[\begin{array}{c} b_{1}\\ b_{2} \end{array}\right]+\left[\begin{array}{cc} Z_{1} & 0\\ 0 & Z_{2} \end{array}\right]\left[\begin{array}{c} a_{1}\\ a_{2} \end{array}\right]+\left[\begin{array}{c} e_{1}\\ e_{2} \end{array}\right],$$

where $y_{1}$ and$\;y_{2}$ are the vectors of phenotypes for trait 1 and trait 2, respectively, $b_{1}\;$and $b_{2}$ are the vectors of fixed effects for two traits, including sex, year, and the covariates of entering weight, fattening days, $a_{1}\sim N\left({0,\;\sigma }_{a_{1}}^{2}G\right)$ and $a_{2}\sim N\left({0,\;\sigma }_{a_{2}}^{2}G\right)$ are the vectors of random additive genetic effects for two traits, where${\;\sigma }_{a_{1}}^{2}$ and $\sigma_{a_{2}}^{2}$ are the additive genetic variance for two traits and G is the additive genetic relationship matrix, X and Z are incidence matrices associating b and a, and $e_{1}\sim N\left({0,\;\sigma }_{e_{1}}^{2}I\right)$ and $e_{2}\sim N\left({0,\sigma }_{e_{2}}^{2}I\right)$ are the vectors of random residuals for two traits, where I is the identity matrix and $\sigma_{e_{1}}^{2}$ and $\sigma_{e_{2}}^{2}$ are the residual variance. The phenotypic and genetic correlation coefficients were calculated using $r_{P}=cov_{P_{12}}/\sqrt{\sigma_{P_{1}}^{2}\times \sigma_{P_{2}}^{2}}$ and$\;r_{G}=cov_{G_{12}}/\sqrt{\sigma_{G_{1}}^{2}\times \sigma_{G_{2}}^{2}}$, where $r_{P}$ and $r_{G}$ are phenotypic and genetic correlation coefficients, respectively, $\sigma_{P_{1}}^{2}$ and $\sigma_{P_{2}}^{2}$ are the phenotypic variance of trait X and trait Y, respectively, $\sigma_{P_{1}}^{2}$ and $\sigma_{P_{2}}^{2}$ are the additive genetic variance of trait 1 and trait 2, respectively, and $cov_{P_{12}}$ and $cov_{G_{12}}$ are the phenotypic and genetic covariance, respectively.

### 2.5. Preselection of Low-Density SNP Panel

Four separate strategies were implemented to select candidate SNPs separately, including genome-wide association study (GWAS), whole-genome regression (BayesB), sliding window, and gene annotation. Before the GWAS and BayesB analyses, the cattle population was split into training (*n* = 1199) and validation (*n* = 132) datasets using birth year before and after 2014, respectively, following the analysis in [9]. Phenotypes of the training dataset were used for variant preselection in GWAS and BayesB analyses for the low-density SNP panel. The phenotypes of the validation dataset were used for the downstream assessment of the predictive performance of the low-density SNP panel. This allowed the validation phenotypes to be independent from the training dataset when predicting direct genomic estimated breeding values (DGVs) for individuals in the validation dataset and allowed analyses to be unbiased. The minor allele frequency (MAF) of 671,204 SNPs after quality control was calculated via PLINK (v1.90), which was used in the sliding window and gene annotation strategies. After completing these four analyses, the candidate SNPs detected by each strategy were merged and deduplicated. Details of the four strategies are described below.

To identify the significantly trait-associated variants, the mixed model-based single locus regression analysis (MMRA) applied to perform GWAS for each trait was as follows:

(2) y=Xβ+Sα+Zμ+e,

where y is a vector of the phenotypes, β is a vector of the fixed effects including the same effects as in Equation (1), α is vector of the fixed genetic effect of a single SNP, μ is a vector of the residual polygenic effect, e is a vector of random residuals, and X, S, and Z are the incidence matrices. Here, we added the top three principal components into β to avoid GWAS being confounded by the population stratification, even if not significant. This analysis was implemented via the R package GenABEL [33]. Lastly, the top 0.1% of SNPs with the lowest *p*-values for each trait were chosen as the potential trait-associated SNPs. 

SNPs contributing to a comparatively large proportion of genetic variance were selected by using whole-genome regression (BayesB), which uses autosomal markers to predict SNP effects simultaneously [8]. BayesB assumes a large number of loci with zero genetic variance and only a small proportion of loci with variance not equal to zero [8]. The statistical model for BayesB is as follows:

(3) y=Xβ+Mg+e,

where y, β, X, and e are as defined in Equation (2), g is the m × 1 vector of the SNP effect, $g_{i}$~$N\left({0,\;\sigma }_{g_{i}}^{2}\right)$, ${\;\sigma }_{g_{i}}^{2}$ is the variance of the i-th SNP effect, and M is the genotype matrix of the training dataset with values equal to 0, 1, or 2. The marker effect variance ${\;\sigma }_{g_{i}}^{2}$ was assigned a prior mixture distribution as follows:

(4) $$\sigma_{g_{i}}^{2}\{\begin{array}{c} =0\;\mathrm{with}\;\mathrm{probability}\;\pi \\ {\sim x}^{-2}\left(v_{g}{,s}_{g}^{2}\right)\;\mathrm{with}\;\mathrm{probability}\;\left(1\;-\;\pi \right) \end{array},\;\mathrm{where}\;i=1,\;\ldots ,\;m.$$

$\sigma_{g_{i}\;}^{2}>\;0$ follows a scaled inverse chi-squared distribution with a probability of (1 − π), where the degrees of freedom is a fixed value (v=4.234) [8] and the scale parameter ($s_{g}^{2}$) is derived from the equation $s_{g}^{2}=\frac{E\left(\sigma_{g_{i}}^{2}\right)\left(v_{g}\;-2\right)}{v_{g}}$. In this study, π was set to 0.999 so that about 100–150 SNPs contributed to additive genetic variance. The Monte Carlo Markov chain (MCMC) algorithm of BayesB consists of running a Gibbs chain. The MCMC chain was run for 50,000 cycles, and the first 10,000 cycles were discarded as burn-in. When obtaining the SNP effects $g_{i}$, the percentage of genetic variance explained by each SNP was calculated by the equation $V_{i}=\frac{{2p}_{i}\left({1\;-\;p}_{i}\right)g_{i}}{\sigma_{a}^{2}}$, where $V_{i}$ is the proportion of additive genetic variance explained by the i-th SNP, $p_{i}$ is the MAF of the i-th SNP, $g_{i}$ is the absolute average substitution effect of the i-th SNP, and $\sigma_{a}^{2}$ is the additive genetic variance. The analysis was implemented via the C language complied software from [27]. The top 0.1% of SNPs were then selected as the affected SNPs. 

Similarly to [20], the highly informative SNPs with a high MAF and uniform spacing across the genome were included by setting a 500 kb sliding window. Specifically, the ARS-UCD1.2 bovine genome assembly was used to define the window over the autosomes to achieve regular spacing, which led to two SNPs being included per Mb. In each window, the SNP with the highest MAF, as well as a call rate >98%, was selected to ensure the polymorphism and robust reproducibility. To avoid a lack of flanking information at the start or end of each chromosome, we included SNP at each chromosomal end.

Given that recent studies have shown that the number of genome features (like genes) that are represented by genetic markers may also influence the performance of GP [34,35], we also added SNPs that were located in each gene region and had high MAF to the low-density SNP panel. The latest bovine genome annotation (Bos_taurus.ARS-UCD1.2) was downloaded from Ensemble (http://asia.ensembl.org/index.html; accessed on 22 October 2019). The SNPs from the BovineHD Beadchip were annotated to the corresponding gene regions (including protein-coding genes, nonprotein-coding genes, and pseudogenes) using bedtools [36]. Consequently, a separate SNP set was formed for each gene, and the SNP with the highest MAF was selected for each set. 

### 2.6. Genomic Prediction

We applied GBLUP [37], BayesA/B [8], and BayesCπ [38] to analyze the performance of the preselected low-density SNP panel for the validation dataset, and the results were compared with that of BovineHD Beadchip using fivefold cross validation.

For GBLUP, the prediction model used to estimate the direct genomic breeding values (DGVs) was basically the same as in Equation (3); however, $g\;\sim N\left({0,\sigma }_{g}^{2}G\right)$ is the vector of breeding values assumed to follow a multivariate normal distribution, where${\;\sigma }_{g}^{2}$ is the genetic variance and G is the genomic relatedness matrix calculated as $G=\frac{\left(M\;-\;P\right){\left(M\;-\;P\right)}^{’}}{2\sum_{i=1}^{m}p_{i}\left({1\;-\;p}_{i}\right)}$. M denotes the (0,1,2) encoded genotype matrix, $p_{i}$ is the MAF of marker i, m denotes the number of markers, and P is a matrix with columns equal to ${2p}_{i}$.

For the three Bayesian approaches, the prediction model used was the same as in Equation (3); however, they used a different prior distribution for $g_{i}$ and prior probability for π. BayesA supposes that each SNP has its own effect; in other words, π = 0. These effects differ from one SNP to another, and the variance $\sigma_{g_{i}}^{2}$ also follows a scaled inverse chi-squared distribution with fixed $v_{g}$ (4.2). For BayesB, the method description was similar to that described above, while the π was set to 0.995 here, since we supposed that candidate QTLs were included in the low-density SNP panel. In comparison with BayesB, the SNP effect variance $\sigma_{g_{i}}^{2}$ in BayesCπ was also assumed a priori to be zero with a probability of π or to follow a scaled inverse chi-squared distribution with a probability of 1 − π, where parameter $v_{g}$ is 4.2. However, each SNP effect has a common variance $\sigma_{g_{i}}^{2}$=$\;\sigma_{g}^{2}$ (j = 1, …, m) in BayesCπ, and π is treated as unknown with a uniform (0,1) prior distribution. The scale parameter ($s_{g}^{2}$) in BayesA/Cπ was also calculated using the above equation. The DGVs were calculated as ${\mathrm{DGV}}_{j}=\sum_{j=1}^{N}Z_{\mathrm{ij}}g_{i}$, where ${\mathrm{DGV}}_{j}$ is the genomic estimated breeding value of the j-th individual of the validation dataset, $g_{i}$ is the estimated effect for the i-th SNP obtained from BayesA/B/Cπ, $Z_{\mathrm{ij}}$ is the encoded genotype of SNP (0/1/2), and N is the number of SNPs. The analyses were conducted using the software from [27].

### 2.7. Assessment of the Low-Density SNP Panel

The number and percentage of markers on each chromosome, and the pairwise marker interval were investigated to ensure the approximately uniform distribution of the low-density SNP panel across the autosomes. The correlation coefficient (*r*^2^) was used to quantify the LD decay of the low-density SNP panel within the 2 Mb window. In addition, to check the feasibility of using low-density marker panels for genotype imputation and genomic prediction of Chinese Simmental beef cattle, we imputed this panel to the ~770 K density using Beagle v4.1 [31]. Prior to this step, the cross-validation scenarios were used to assess the accuracy of imputation, wherein different groups of animals were included in the reference population to predict the DGVs of animals in validation set. The Chinese Simmental beef cattle were split at random into five near-equal subsets. At each cross-validation rotation, the reference and validation sets included around 1076 (four subsets) and 260 animals (one subset), respectively. The accuracy of imputation was taken as the proportion of genotypes that were correctly imputed. This process was then repeated five times.

For the low-density SNP panel, the prediction accuracy was assessed only for the validation datasets that included individuals born after 2014. For BovineHD Beadchip, the accuracy of genomic prediction was assessed using fivefold cross-validation (CV), which assigned animals randomly into five separate subsets of near-equal size. Each subset was used as the validation set only once, with the phenotype masked, while the remaining four subsets were treated as the training set. To reduce random sampling effects, the CV layout described above was replicated 20 times, and a new randomization was implemented for each replicate so that each of the subsets contained different individuals. The prediction accuracies were calculated as the correlation between the DGVs and the adjusted phenotypes in the validation set divided by the square root of the heritability. In addition, to assess the extent of bias of genomic prediction, the linear regression coefficients (b (y, DGV)) of the adjusted phenotypes (y) on the DGVs were calculated for the validation dataset. Here, phenotypes were adjusted for the environmental fixed effects, including sex, year, and the covariates of body weight upon entering the fattening farm, and the number of fattening days, and the residuals were saved as the adjusted phenotype. Unbiased models should not significantly deviate from 1, whereas values greater than 1 indicate a biased deflation prediction of DGVs and values smaller than 1 indicate a biased inflation prediction of DGVs.

## 3. Results

### 3.1. Statistics and Population Structure

**Figure 1:**
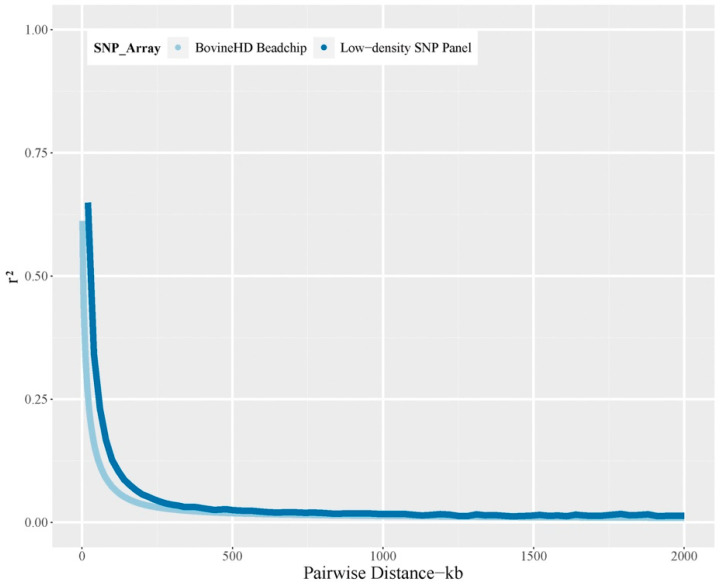
The linkage disequilibrium (*r*^2^) decay patterns for the low-density SNP panel and Illumina BovineHD beadchip within the 2 Mb window.

The statistical descriptions for each trait are summarized in Table 1. Regarding the population structure of the Chinese Simmental beef cattle population, results showed that this population could be separated into five clusters on the basis of the first two principal components (Figure S1, Supplementary Materials); however, many individuals were clustered together. The linkage disequilibrium (LD) of this population was quantified via the *r*^2^ value with the 770 K chip after quality control. We found that the LD decreased from 0.61 to 0.01 within the 2 Mb window (Figure 1). The LD dropped below 0.2 at distances of 34 kb and remained steady when the SNP interval increased to 500 kb. 

### 3.2. Estimation of Genetic Parameters

**Figure 2:**
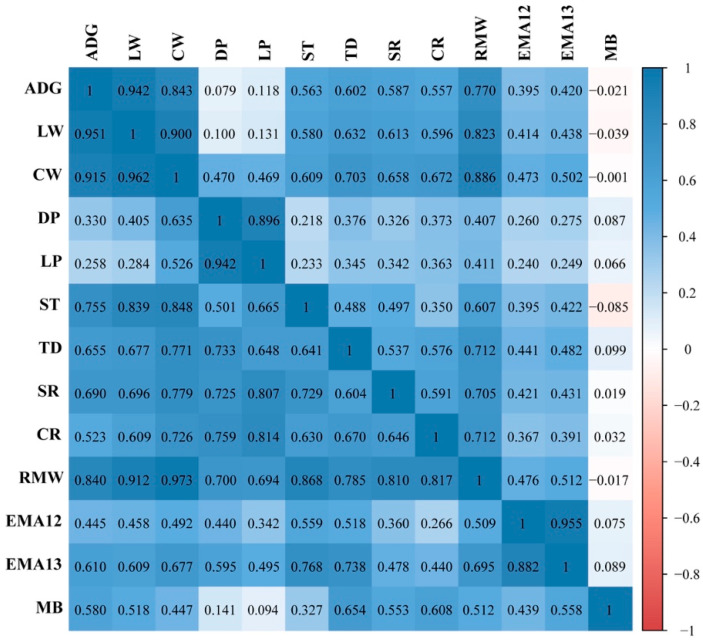
The estimated genetic and phenotypic correlations of 13 traits. The upper and lower triangle regions of the matrix show the phenotypic correlations and genetic correlations, respectively.

The heritability value of the 13 traits ranged from 0.11 to 0.56 (Table 1). Among these traits, we found six traits had relatively high heritability, namely, CR (0.56), CW (0.42), ST (0.41), RMW (0.39), TD (0.39), SR (0.39), and LW (0.38), and four traits displayed moderate heritability, namely, LP (0.35), DP (0.29), EMA13 (0.28), and CR (0.28); the remaining two traits, EMA12 (0.18) and MB (0.11), had low heritability (Table 1). Generally, growth and carcass traits had moderate to high heritability. The genetic correlations (below diagonal) and phenotypic correlations (above diagonal) are presented in Figure 2. The genetic correlation for all pairwise traits was positive and ranged from 0.7 to 0.8. Nevertheless, negative phenotypic correlations could be observed in the pairwise traits, and almost all phenotypic correlations were lower than the corresponding genetic correlations. Among all traits, two growth traits had high genetic correlations with most carcass traits and had moderate genetic correlations with meat quality traits. A similar pattern was also observed for phenotypic correlations.

### 3.3. Features of the Low-Density SNP Panel

After the implementation of a genome-wide association study for 13 traits, SNPs ranking in the top 0.1% were selected as trait-associated loci. A total of 671,204 SNPs from the BovineHD Beadchip were kept after quality control. Approximately, 672 SNPs ranking in the top 0.1% were selected for each trait. This contributed to a total of 6932 SNP was included after filtering, with 1804 overlapping SNPs detected in at least two traits. The GWAS results of 13 traits are summarized in Figure S2 (Supplementary Materials). Similarly, 6421 SNPs that explained a comparatively large proportion of the genetic variance were screened (672 for each trait) after omitting 2315 overlapping SNPs. In terms of the informative SNPs with a high MAF, 5087 SNPs were kept by setting a 500 kb sliding window across the genome. In addition, 16,286 genes entries of the reference genome were annotated for the SNPs of BovineHD Beadchip, which accounted for 66.30% of the total genes in the reference genome. Chromosomes 3, 7, 19, 5, 18, and 11 had comparatively large proportions of the total gene entries, with values of 6.3%, 6.1%, 5.9%, 5.8%, 5.5%, and 4.7%, respectively (Table S1, Supplementary Materials). The SNP with the highest MAF value in each gene entry was screened out. Lastly, a low-density SNP panel consisting of 30,684 SNPs was formed by merging together all preselected SNPs from the above analyses and deduplicating them (Table S2, Supplementary Materials). 

Generally, SNPs in the low-density SNP panel had an approximately uniform distribution across autosomes, with 10–20 SNPs per 1 Mb window (Figure S3, Supplementary Materials), which was in accordance with the pattern showing that the number of SNPs in the autosomes decreased as the length was reduced. In comparison with BovineHD Beadchip, the percentage of SNPs from the low-density SNP panel increased in chromosomes 3, 5, 7, 11, 13, 15, 18, and 19 (Table S3, Supplementary Materials). The trait-associated SNPs identified by GWAS were mainly clustered in above chromosomes. For example, with respect to carcass traits, SNPs with low *p*-values were detected in chromosomes 5, 7, and 11 (Figure S1, Supplementary Materials). Analogously, the whole-genome regression found that the loci that contributed to the large proportion of the genetic variance were in chromosomes 5, 7, and 11. Approximately 6% of the total SNPs characterized by GWAS and BayesB were enriched in chromosome 11. In addition, many SNPs in these chromosomes were selected on the basis of gene annotation strategies (Table S1, Supplementary Materials). The mean and median values of the SNP interval of the low-density SNP panel were 81.8 kb and 37.7 kb, respectively, of which 88.6% of the SNP interval was less than 200 kb and only 1.5% of the SNP interval was greater than 500 kb (Figure S3, Supplementary Materials). Regarding the MAF of this panel, the mean and median value were 0.35 and 0.39, respectively (Figure S3, Supplementary Materials). The linkage disequilibrium (LD) of the low-density SNP panel was investigated within the 2 Mb window (Figure 1), within which this ~30 K panel had a similar LD decay pattern to the BovineHD Beadchip. When the physical distance of the SNPs reached 2 Mb, the LD of the low-density SNP panel dropped from 0.65 to 0.01, while the LD of BovineHD Beadchip analogously decreased from 0.61 to 0.01. Both LD values remained steady when the SNP intervals increased to 500 kb, despite the slightly faster decay of LD in the BovineHD Beadchip. When imputing the low-density SNP panel to the high-density level, the accuracy of imputation was similar across the five cross-validations, with an average accuracy level of 97.4%, indicating that the ~30 K panel is applicable for the cost-effective GP in Chinese Simmental beef cattle.

### 3.4. Prediction Accuracy of Low-Density SNP Panel

The prediction accuracy of DGVs for both SNP panels was evaluated using GBLUP and BayesA/B/Cπ (Table 2). For the low-density SNP panel, the predictive accuracies of the 13 traits ranged from 0.23 to 0.47 in GBLUP, and from 0.22 to 0.47, 0.23 to 0.43, and 0.23 to 0.44 in BayesA/B/Cπ, respectively. For the BovineHD Beadchip, the prediction accuracies of the 13 traits were 0.21–0.61, 0.24–0.53, 0.23–0.53, and 0.22–0.54 in GBLUP and BayesA/B/Cπ, respectively. For both SNP panels, RMW had the highest accuracy across four methods; two growth traits (ADG and LW) had an approximately average accuracy of 0.40; carcass traits (RMW, CR, SR, TD, ST, LP, and DP) had comparatively high prediction accuracy, with an average value higher than 0.3, while the accuracies of meat quality traits were lower than other two types of trait, with an average value of about 0.3. When comparing the accuracy of the low-density SNP panel with that of BovineHD Beadchip, the accuracies for ST, RMW, SR, DP, ADG, CR, and LP decreased by 0.20, 0.09, 0.09, 0.07, 0.05, 0.04, and 0.03 on average, respectively. Notably, most traits showed a decrease in accuracy of less than 0.07, except for ST and RMW. Nevertheless, the use of a low-density SNP panel led to improvements in accuracies for some traits; specifically, the accuracies of LW, TD, EMA12, EMA13, and MB were improved by 0.06, 0.04, 0.06, 0.06, and 0.07 on average, respectively.

### 3.5. Regression Coefficients of the Low-Density SNP Panel Using Different Methods

Table 3 displays the phenotype regression on DGVs. For the low-density SNP panel, the regression coefficients of the 13 traits were 0.764–1.372, 0.928–1.561, 1.199–1.430, and 0.790–1.367 for GBLUP and BayesA/B/Cπ, respectively. Except for the coefficients of ADG, TD, and CR, the values showed significant deviation from 1, while MB showed the highest bias across four methods, suggesting that the level of bias of the genomic predictions for these traits increased. For BovineHD Beadchip, the regression coefficients for ADG, LW, DP, LP, TD, SR, and RMW were not significantly different from 1 for any of the methods, indicating that the predictions were not significantly biased, while the regression coefficients for EMA12, EMA13, and MB were significantly different from 1 for the four methods. When comparing the low-density SNP panel to BovineHD Beadchip, we found that the latter’s regression coefficients were closer to 1, with less bias in prediction, especially for LP, DP, CW, MB, and EMA12.

### 3.6. Prediction Performance of Different Methods

**Figure 3:**
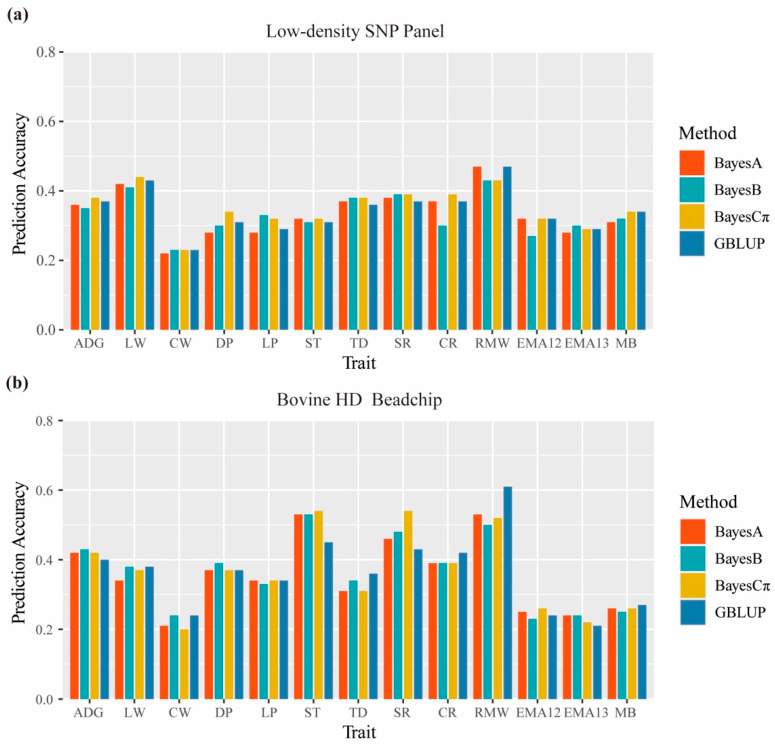
(**a**) The prediction accuracy of DGVs for the low-density SNP panel using BayesA, BayesB, BayesCπ, and GBLUP methods. (**b**) The prediction accuracy of DGVs for the Illumina BovineHD using BayesA, BayesB, BayesCπ, and GBLUP methods.

When using the low-density SNP panel, BayesCπ displayed the best predictive performance, followed by GBLUP, BayesA, and BayesB (Figure 3). The accuracies of ADG, LW, CW, DP, ST, SR, and CR were higher for BayesCπ than for the other three methods. GBLUP outperformed BayesB and BayesA in terms of ADG, LW, CW, DP, and RMW, while BayesA delivered better predictive performance than BayesB in terms of ADG, LW, ST, CR, RMW, and EMA12. In the BovineHD Beadchip, Bayesian methods performed better than GBLUP for ADG, DP, ST, SR, EMA12, and EMA13, and the accuracies of ST and SR estimated by Bayesian methods were 0.08 and 0.06 higher, respectively, than those of GBLUP on average. However, the prediction accuracies of CW, LW, TD, CR, and RMW using GBLUP were higher than those of Bayesian methods, especially in terms of RMW, for which the accuracy of GBLUP was 0.08, 0.11, and 0.09 higher than that of BayesA, BayesB, and BayesCπ, respectively. On the other hand, when comparing the regression coefficients of the four methods with each other, we found that GBLUP performed better than the three Bayesian methods in both SNP panels, with the regression coefficients being closer to 1 for most traits, indicating that GBLUP contributed to less bias in the genomic predictions. Of the three Bayesian methods, BayesCπ delivered better predictive performance than BayesA and BayesB in the low-density SNP panel, since the deflation of the DGVs predicted by it was smaller.

## 4. Discussion

### 4.1. The Selection Strategies of the Low-Density SNP Panel 

Compared with previous studies that merely focused on developing a low-density SNP chip for imputation or a trait-specific low-density chip, we took both purposes into account to design a customized low-density SNP panel for the Chinese Simmental beef cattle. An approximate 97% imputation accuracy could be obtained when imputing this panel for the 770 K. The high imputation accuracy could be attributed to the selection of the SNPs with high MAF, whereby the low-density SNPs could reflect the genome polymorphism of the Chinese Simmental beef cattle. Similarly, a previous study used the same strategy to form a low-density SNP chip for GP and achieved over 97% imputation accuracy when imputed it to Illumina BovineSNP50 [20].

Compared with the other studies that used whole genome-wide association studies (GWAS) [9] or Bayesian GWAS [22] to screen the candidate variants of one trait for low-density SNP chip, our study used both methods to select potential causal variants of 13 traits. As expected, our panel achieved moderate to high prediction accuracy across all traits, which was beneficial for the inclusion of these variants since the effect of causal variants could be estimated directly and the non-associated variants that may have diluted the true genetic signals were removed [39]. Previously, researchers did not take the genome annotation into account when designing a low-density SNP panel. Recent studies on genomic prediction showed that incorporating the genome annotation [40,41] into GP can improved the prediction accuracy. Our recent study integrating the gene annotation into prediction models demonstrated that the number of gene entries that are represented by the SNP has an impact on the prediction accuracy [42]. To make the low-density SNP panel reflect more gene entries, our study included SNPs that have the highest MAF in each gene entry. 

### 4.2. Estimation of Genetic Parameters

In our study, heritability estimates ranging from low to high (from 0.11 to 0.56) were generated for 13 traits in Chinese Simmental beef cattle. The estimates for LW, CW, and RMW in this study were similar to those in a previous study on Chinese Simmental beef cattle, while the estimates for DP, LP, ST, and SR were 0.12, 0.21, 0.16, and 0.13 higher, respectively, than those found previously [26], which was partially due to the comparatively larger reference population used for the variance component estimations in our study. Comparing our results with previously reported estimates for other cattle populations, we found that the estimated heritability levels of CW (0.42) and ADG (0.37) were consistent with those from reports on American Angus (CW, 0.40) [43] and Multibreed cows (ADG; 0.34) [44], but were lower or higher than those found in Simmental (CW, 0.48 [45]), Japanese Black (CW, 0.56) [46], Hanwoo (CW, 0.33 [47]), and Nellore (CW, 0.17 [48]; ADG,0.31 [49]) cattle breeds. The heritability levels for EMA13 (0.28) and MB (0.11) were below the corresponding values found above for Japanese Black (0.42 and 0.56) and Hanwoo (0.37 and 0.40) cattle populations, respectively. The reasons for such differences in heritability were the different population scales and genomic relationship matrices used for heritability estimations. Among all traits, growth traits had a high genetic correlation with most carcass traits, but had a moderate genetic correlation with meat quality traits (Figure 2). The genetic correlations between ADG and LW, CW, ST, TD, and MB were consistent with a previous report on Chinese Simmental beef cattle [50].

### 4.3. The Prediction Accuracy of the Low-Density SNP Panel 

The usage of the low-density SNP panel achieved moderate to high accuracy in studied traits, even though it decreased the prediction accuracies in most of the traits in comparison with those using the BovineHD Beadchip. Among these 13 traits, ST, RMW, and SR displayed the greatest decreases, with average decreases of 0.20, 0.09, and 0.09 across the four methods, respectively; however, DP, LP, CR, and ADG exhibited slight decreases ranging from 0.02 to 0.07. These losses in accuracy were mainly attributed to the decrease in marker density in the low-density SNP panel, since the prediction accuracy should, in theory, be positively associated with the marker density [9,13]. The marker density dropping from 770 K to 30 K may increase the physical distance between QTLs and markers and reduce linkage disequilibrium (LD) between them. The imperfect LD between them resulted in markers not explaining more genetic variance on behalf of QTLs. The prediction accuracy decreases quickly if the linkage between the markers and QTLs is low [51]. 

In contrast to the decrease in accuracy for the above seven traits, the accuracy of LW prediction using the low-density SNP panel was very close to that using the BovineHD Beadchip, and even the accuracies of LW, TD, EMA12, EMA13, and MB benefited from the usage of the low-density SNP panel, with average accuracy increases of 0.04–0.07. These unexpected improvements indicated that the low-density SNP panel containing candidate QTLs and informative SNPs for a low-density SNP panel could be feasible and useful for cost-effective prediction, even if the marker density is much smaller. Our study suggested that the genetic variances of these five traits were determined by a small number of variants with large effects. The selection of SNPs associated with traits or explaining a great percentage of the genetic variance allowed the QTL effects to be directly captured by the models [52]. The high average identical by state (IBS = 0.65) value between the training and validation datasets, as well as the small effective population size (Ne = 74 estimated by [53]) of this breeding population, could have resulted in an extensive LD and a smaller number of effective chromosome segments being estimated. This, in turn, may have advantaged the high prediction accuracy of these traits using the low-density SNP panel. 

### 4.4. The Prediction Accuracy of Four Prediction Methods

To allow a more reliable assessment of the predictive performance of the low-density SNP panel, four methods with different model assumptions were applied. No consensus exists to date on the best genomic prediction tool, in spite of plenty of comparisons having been made for various prediction methods in both simulations [7,52] and real datasets [26,43]. In our study, the BayesCπ model outperformed the others for most traits, indicating that BayesCπ successfully interprets the genetic architecture of these traits. The accuracy of GEBV relies on the consistency between the assumption distribution of the locus effects and the true pattern [54]. Unlike BayesA and BayesB, which have a great sensitivity to the assumption of the locus effects, the prior value of BayesCπ is inferred from real circumstances [55]. In BayesCπ, the posterior value of the π of the 13 traits ranged from 0.78–0.96, lower than the fixed value for BayesB (0.995). The performance of GBLUP was worse than that of BayesCπ, which may be attributed to the inferior capture of the genetic relationships between cattle using the low-density SNP panel. Similar results have also been reported from studies that analyzed the impacts of marker density on GP for both real [56] and simulation datasets [57]. Regarding the BovineHD Beadchip, the predictive performance of the Bayesian models was superior to that of the GBLUP, which was in line with the results of a previous study on the same cattle population [26]. 

### 4.5. Application of the Low-Density SNP Panel 

The customized low-density SNP panel will be applied for the cost-effective GP of the 13 traits of the Chinese Simmental beef cattle. The calves will be genotyped by the low-density SNP panel, and the genomic estimated breeding value (GEBV) will be calculated. Animals with GEBV ranking in the top 10% will be selected as candidates for breeding purposes. Compared to the prediction accuracy of the BovineHD Beadchip, the accuracy of the low-density SNP panel was slightly lower. For a more accurate estimation of genetic merit and candidate selection, the calves passing the first step selection will be genotyped by the BovineHD Beadchip. The two-step genotyping will greatly reduce the genotyping costs by avoiding BovineHD Beadchip genotyping for all animals. Meanwhile, the second step of genotyping can ensure the satisfactory prediction accuracy of GEBV and the selection of breeding calves. In this setting, we assume that the low-density SNP panel would be a feasible and applicable way for cost-effective genomic prediction in Chinese Simmental beef cattle. The size of the reference population, as well as the genetic relationship between the training and validation datasets, plays a key role in genomic prediction. In future, the low-density SNP panel will be updated as the number of animals in the reference population increases. For example, new variants with larger estimated effects will be included and less informative variants in the current panel with small effects will be removed, while the marker density of the low-density SNP panel will be kept at approximately 30 K. With the availability of whole-genome sequencing of the Chinese Simmental cattle, the SNP list of the SNP panel will be refined to enhance the prediction power. The causal variants of 13 traits identified using the whole-genome sequencing data will also added to the panel in the near future. 

## 5. Conclusions

A customized low-density SNP panel with 30,684 informative markers was developed and used to predict useful genomic estimated breeding values in 13 growth, carcass, and meat quality traits of Chinese Simmental beef cattle, aiming to reduce breeding costs and support the application of genomic prediction. To enable comparison, the prediction accuracies using BovineHD Beadchip were treated as references. We found that the performance of the ~30 K SNP genotyping array was trait-dependent; it reduced the predictive accuracies of seven traits but improved the accuracies of five traits. While differences in terms of prediction accuracy were observed among the 13 traits, the low-density SNP panel achieved moderate to high accuracy for most of the traits and even improved accuracy for some traits. Overall, the low-density SNP panel (~30 K) is a feasible and promising tool for cost-effective genomic prediction in Chinese Simmental beef cattle when only one or a few key economic traits are of interest.

## Figures and Tables

**Table 1:** Statistics and estimated heritability for studied traits in Chinese Simmental beef cattle.

Traits ^1^		The Number of Phenotypes	Mean (SD)	*h*^2^ (SE)	$\sigma_{a}^{2}\;$	$\sigma_{e}^{2}\;$
Growth traits	ADG	1330	0.96 ± 0.22	0.37 ± 0.06	0.12	0.17
	LW	1342	504.95 ± 70.22	0.38 ± 0.07	4586.61	7483.41
Carcass traits	CW	1346	270.67 ± 45.20	0.42 ± 0.05	314.05	433.69
	DP	1341	53.56 ± 2.91	0.28 ± 0.06	2.04	5.23
	LP	1338	45.47 ± 3.00	0.35 ± 0.07	3.00	5.57
	ST	1342	8.55 ± 1.99	0.40 ± 0.05	0.75	1.13
	TD	1341	3.97 ± 0.70	0.28 ± 0.07	2.04	5.24
	SR	1341	10.57 ± 2.23	0.39 ± 0.07	0.12	0.19
	CR	1334	11.47 ± 3.25	0.56 ± 0.06	1.98	1.56
	RMW	1344	167.79 ± 30.15	0.39 ± 0.07	112.42	175.83
Meat quality traits	EMA12	1343	85.53 ± 13.58	0.18 ± 0.06	21.20	96.59
	EMA13	1203	85.21 ± 14.13	0.28 ± 0.06	26.19	67.35
	MB	1343	5.14 ± 1.00	0.11 ± 0.05	0.27	2.18

^1^ Growth traits: average daily gain (ADG; kg) and live weight (LW; kg). Carcass traits: hot carcass weight (CW; kg), dressing percentage (DP; %), lean meat percentage (LP; %), weight of retail beef cuts including striploin (ST; kg), spencer roll (SR; kg), chuck roll (CR; kg), and tenderloin (TD; kg), and retail meat weight (RMW). Meat quality traits: eye muscle area at the 12th rib (EMA12), eye muscle area at the 13th rib (EMA13), and marbling at the 12th rib (MB).

**Table 2:** Predictive accuracy of GEBVs for the low-density SNP panel using different methods.

Traits ^1^		GBLUP		BayesA		BayesB		BayesCπ	
		LD ^2^	HD ^3^ (SD)	LD	HD (SD)	LD	HD (SD)	LD	HD (SD)
Growth traits	ADG (−0.05)	0.37	0.40 (0.06)	0.36	0.42 (0.06)	0.35	0.43 (0.06)	0.38	0.42 (0.06)
	LW (+0.06)	0.43	0.38 (0.05)	0.42	0.34 (0.06)	0.41	0.38 (0.06)	0.44	0.37 (0.06)
Carcass traits	CW (0)	0.23	0.24 (0.06)	0.22	0.21 (0.06)	0.23	0.24 (0.06)	0.23	0.20 (0.06)
	DP (−0.07)	0.31	0.37 (0.05)	0.28	0.37 (0.06)	0.30	0.39 (0.06)	0.34	0.37 (0.06)
	LP (−0.03)	0.29	0.34 (0.06)	0.28	0.34 (0.05)	0.33	0.33 (0.06)	0.32	0.34 (0.06)
	ST (−0.20)	0.31	0.45 (0.06)	0.32	0.53 (0.06)	0.31	0.53 (0.06)	0.32	0.54 (0.06)
	TD (+0.04)	0.36	0.36 (0.05)	0.37	0.31 (0.06)	0.38	0.34 (0.06)	0.38	0.31 (0.06)
	SR (−0.09)	0.37	0.43 (0.06)	0.38	0.46 (0.06)	0.39	0.48 (0.05)	0.39	0.54 (0.05)
	CR (−0.04)	0.37	0.42 (0.05)	0.37	0.39 (0.06)	0.30	0.39 (0.06)	0.39	0.39 (0.06)
	RMW (−0.09)	0.47	0.61 (0.06)	0.47	0.53 (0.07)	0.43	0.50 (0.06)	0.43	0.52 (0.06)
Meat quality traits	EMA12 (+0.06)	0.32	0.24 (0.07)	0.32	0.25 (0.07)	0.27	0.23 (0.07)	0.32	0.26 (0.07)
	EMA13 (+0.06)	0.29	0.21 (0.07)	0.28	0.24 (0.07)	0.30	0.24 (0.07)	0.29	0.22 (0.07)
	MB (+0.07)	0.34	0.27 (0.07)	0.31	0.26 (0.07)	0.32	0.25 (0.07)	0.34	0.26 (0.07)

^1^ Traits with an average decrease or increase in prediction accuracy. Growth traits: average daily gain (ADG; kg) and live weight (LW; kg). Carcass traits: hot carcass weight (CW; kg), dressing percentage (DP; %), lean meat percentage (LP; %), weight of retail beef cuts including striploin (ST; kg), spencer roll (SR; kg), chuck roll (CR; kg), and tenderloin (TD; kg), and retail meat weight (RMW). Meat quality traits: eye muscle area at the 12th rib (EMA12), eye muscle area at the 13th rib (EMA13), and marbling at the 12th rib (MB). ^2^ Prediction accuracy of the low-density SNP panel. ^3^ Prediction accuracy with standard deviation of BovineHD Beadchip; prediction accuracies were averaged over the fivefold cross-validation (CV) and then over the 20 replicates in BovineHD Beadchip.

**Table 3:** Regression coefficients for GEBVs for the low-density SNP panel using different methods.

Traits ^1^		GBLUP		BayesA		BayesB		BayesCπ	
		LD ^2^	HD ^3^	LD	HD	LD	HD	LD	HD
Growth traits	ADG	0.914	0.989	1.397	1.022	1.198	0.970	1.198	1.039
	LW	1.175	1.011	1.300	0.971	1.342	0.926	1.150	1.045
Carcass traits	CW	0.812	1.102	0.928	0.967	1.198	1.202	0.852	1.082
	DP	0.922	1.075	1.379	0.976	1.331	1.057	1.022	1.151
	LP	0.904	0.963	1.343	0.910	1.323	1.041	1.008	1.025
	ST	1.023	1.064	1.387	1.225	1.210	1.082	1.210	1.077
	TD	1.106	1.064	1.389	1.056	1.271	0.974	1.201	1.082
	SR	1.094	1.059	1.388	1.093	1.242	0.993	1.158	0.965
	CR	0.923	1.040	1.170	1.092	1.199	1.148	1.199	1.094
	RMW	1.164	1.039	1.203	0.944	1.234	1.031	1.257	0.991
Meat quality traits	EMA12	0.924	1.116	1.460	0.983	1.263	0.958	0.937	1.082
	EMA13	0.764	1.025	1.419	1.117	1.293	1.167	0.790	1.142
	MB	1.372	1.125	1.561	1.122	1.430	1.210	1.367	1.159

^1^ Growth traits: average daily gain (ADG; kg) and live weight (LW; kg). Carcass traits: hot carcass weight (CW; kg), dressing percentage (DP; %), lean meat percentage (LP; %), weight of retail beef cuts including striploin (ST; kg), spencer roll (SR; kg), chuck roll (CR; kg), of tenderloin (TD; kg), and retail meat weight (RMW). Meat quality traits: eye muscle area at the 12th rib (EMA12), eye muscle area at the 13th rib (EMA13), and marbling at the 12th rib (MB). ^2^ Regression coefficients of the low-density SNP panel. ^3^ Regression coefficients with standard deviation of the BovineHD Beadchip; regression coefficients were averaged over the fivefold cross-validation (CV) and then over the 20 replicates in BovineHD Beadchip.

## Data Availability

Genotype data were submitted to Dryad: doi:10.5061/dryad.4qc06. The bovine genome annotation (Bos_taurus.ARS-UCD1.2) was downloaded from Ensemble.

## Supplementary Materials

The following are available online at https://www.mdpi.com/article/10.3390/ani11071890/s1: Figure S1. The principal component analysis of the Chinese Simmental beef cattle; Figure S2. The results of genome-wide association study for 13 traits of Chinese Simmental beef cattle; the black line in the Manhattan plot represents the Bonferroni corrected threshold of 7 × 10^−8^(0.05/671,204); Figure S3. (a) SNP distribution of the low-density SNP panel across autosomes. (b) Probability density of SNPs interval of the low-density SNP panel. (c) Minor allele frequency (MAF) distribution of the low-density SNP panel in Chinese Simmental beef cattle; Table S1. The number and percentage of genes represented by the SNPs of the BovineHD Beadchip across autosomes; Table S2. The number of SNPs selected using different strategies; Table S3. The number and percentage of SNPs on each autosome included in the low-density SNP panel and BovineHD Beadchip.
