# Supplementary material for: Validation of the Prediction Accuracy for 13 Traits in Chinese Simmental Beef Cattle Using a Preselected Low-Density SNP Panel
Source: Animals (Basel). 2021 Jun 25;11(7):1890. doi: 10.3390/ani11071890 (PMC8300368; doi:10.3390/ani11071890)
Supplement: Supplementary file 1 [file animals-11-01890-s001.zip › Supplementary Figure legends.pdf]

Figure S1. The principal component analysis of the Chinese Simmental beef cattle.
Figure S2. The results of genome-wide association study for 13 of Chinese Simmental beef cattle; the black line in the Manhattan-plot represents the Bonferroni corrected threshold of 7.00E-08 (0.05/671,204).
Figure S3. (a) SNP distribution of the low-density SNP panel across autosomes. (b) Probability density of SNPs interval of the low-density SNP panel. (c) Minor allele frequency (MAF) distribution of the low-density SNP panel in Chinese Simmental beef cattle.
