# Supplementary material for: Validation of the Prediction Accuracy for 13 Traits in Chinese Simmental Beef Cattle Using a Preselected Low-Density SNP Panel
Source: Animals (Basel). 2021 Jun 25;11(7):1890. doi: 10.3390/ani11071890 (PMC8300368; doi:10.3390/ani11071890)
Supplement: Supplementary file 1 [file animals-11-01890-s001.zip › Supplementary tables.pdf]

Table S1. The number and percentage of genes represented by the SNPs of the BovineHD Beadchip across autosomes.

Chromosome	The number of genes[1]	The percentage of genes (%)[2]
Chr1	687	4.22
Chr2	722	4.43
Chr3	1,020	6.26
Chr4	572	3.51
Chr5	945	5.8
Chr6	481	2.95
Chr7	991	6.09
Chr8	547	3.36
Chr9	391	2.4
Chr10	737	4.53
Chr11	768	4.72
Chr12	281	1.73
Chr13	632	3.88
Chr14	359	2.2
Chr15	677	4.16
Chr16	512	3.14
Chr17	464	2.85
Chr18	900	5.53
Chr19	953	5.85
Chr20	258	1.58
Chr21	401	2.46
Chr22	449	2.76
Chr23	533	3.27
Chr24	237	1.46
Chr25	564	3.46
Chr26	312	1.92
Chr27	170	1.04
Chr28	242	1.49
Chr29	481	2.95
Total	16,286	100

[1] The number of genes that was annotated by the SNPs of the BovineHD Beadchip for each autosome.

[2] The percentage of genes that was annotated by the SNPs of the BovineHD Beadchip for each autosome.

Table S2. The number of SNP selected by different strategies. # The number of

Method	# SNPs selected by each method [1]	# SNPs after removing duplicate SNP in each method 2	# SNPS after removing duplicate SNP between methods [3]
GWAS	8736	6932	-
BayesB	8736	6421	-
Sliding window	5087	5087	-
Genome Annotation	16286	16286	-
Total	38845	34726	30,684

Table S3. The number and percentage of SNPs on each autosome included in the Low-density SNP Panel and BovineHD Beadchip

Chromosome	Low-density SNP Panel		BovineHD Beadchip[1]	
	The number of SNPs	The percentage of SNPs (%)	The number of SNPs	The percentage of SNPs (%)
Chr1	1,675	5.46	42,222	6.29
Chr2	1,374	4.48	36,411	5.42
Chr3	1,651	5.38	32,683	4.87
Chr4	1,307	4.26	32,168	4.79
Chr5	1,538	5.01	31,561	4.70
Chr6	1,186	3.87	32,396	4.83
Chr7	1,641	5.35	30,291	4.51
Chr8	1,207	3.93	28,769	4.29
Chr9	857	2.79	27,912	4.16
Chr10	1,368	4.46	28,293	4.22
Chr11	1,543	5.03	30,055	4.48
Chr12	788	2.57	23,486	3.50
Chr13	1,219	3.97	20,792	3.10
Chr14	942	3.07	21,153	3.15
Chr15	1,267	4.13	22,852	3.40
Chr16	970	3.16	22,136	3.30
Chr17	824	2.69	20,415	3.04
Chr18	1,187	3.87	18,098	2.70
Chr19	1,294	4.22	17,661	2.63
Chr20	624	2.03	20,132	3.00
Chr21	927	3.02	19,290	2.87
Chr22	674	2.20	16,950	2.53
Chr23	813	2.65	14,099	2.10
Chr24	567	1.85	16,914	2.52
Chr25	871	2.84	12,095	1.80
Chr26	617	2.01	14,280	2.13
Chr27	442	1.44	12,308	1.83
Chr28	550	1.79	12,130	1.81
Chr29	761	2.48	13,652	2.03
Total	30,684	100	671,204	100

[1] The BovineHD Beadchip after quality control.
