# Supplementary figures and images for: Validation of the Prediction Accuracy for 13 Traits in Chinese Simmental Beef Cattle Using a Preselected Low-Density SNP Panel
Source: Animals (Basel). 2021 Jun 25;11(7):1890. doi: 10.3390/ani11071890 (PMC8300368; doi:10.3390/ani11071890)

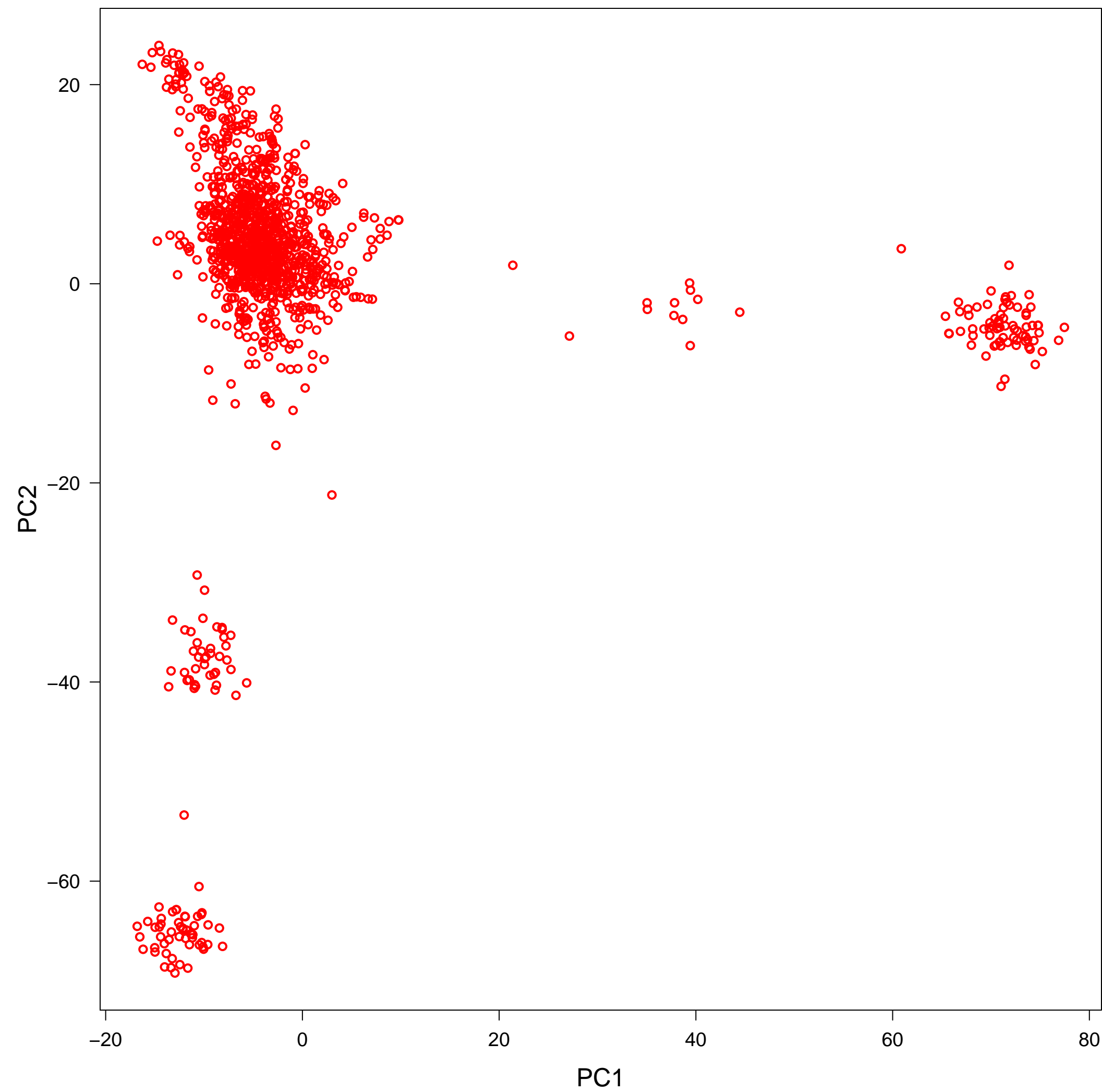

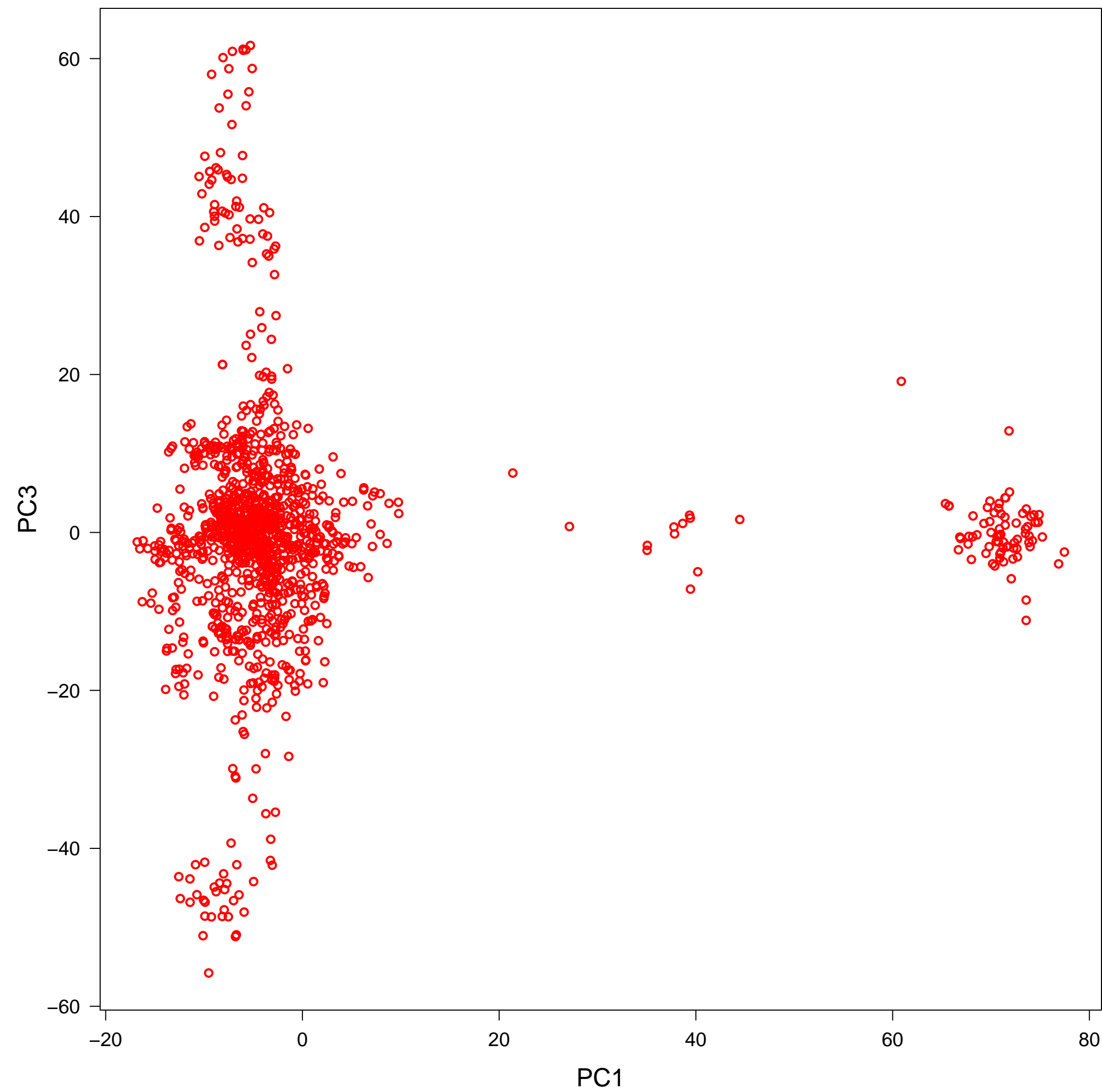

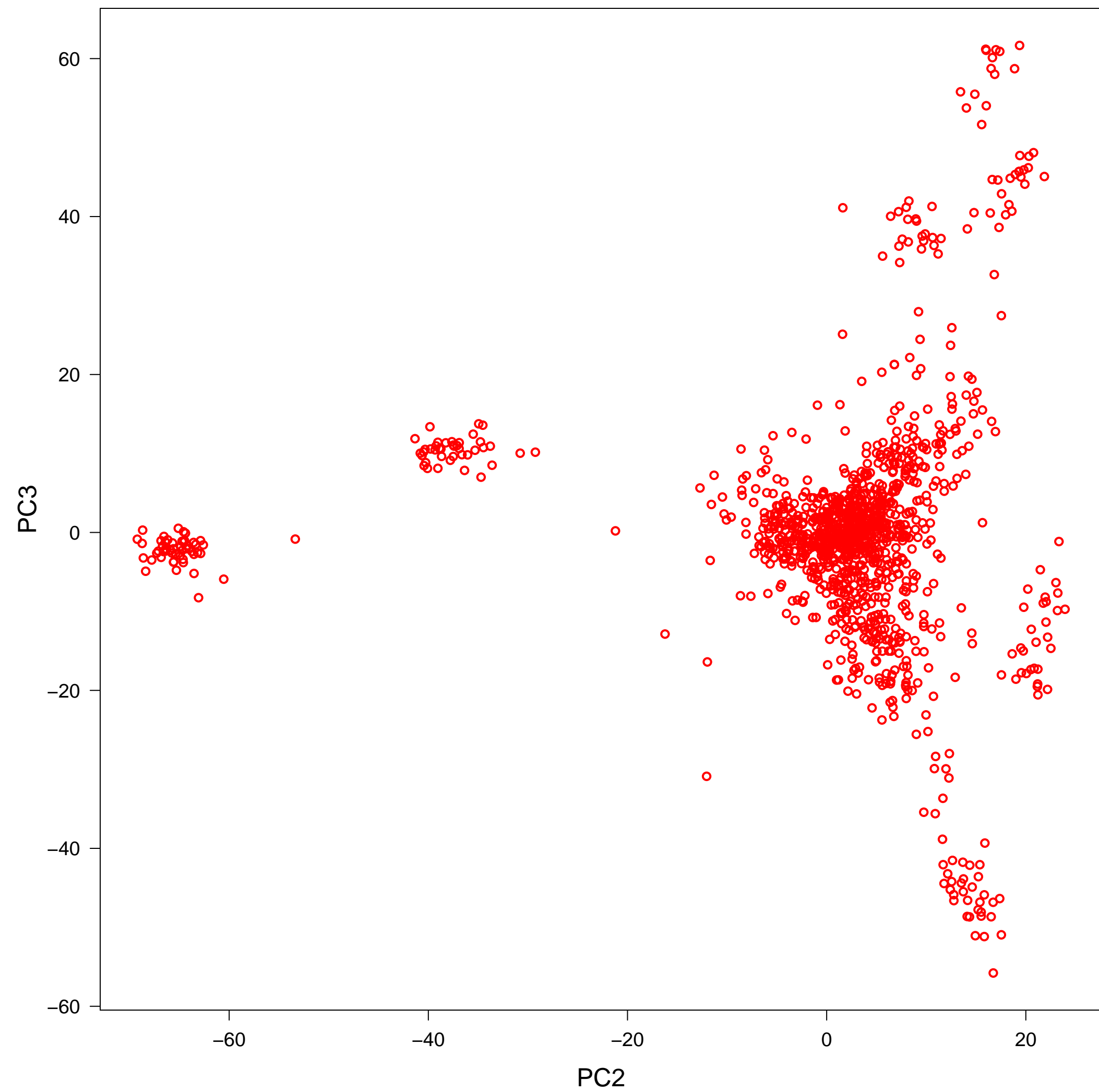

Supplement: Supplementary file 1 [file animals-11-01890-s001.zip › Figure S1.pdf]

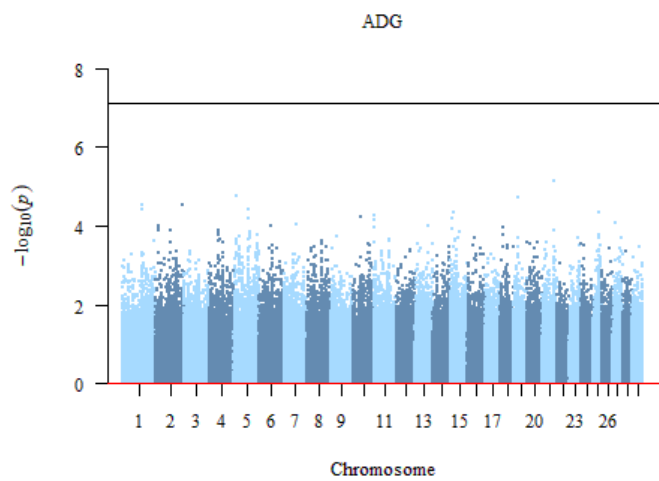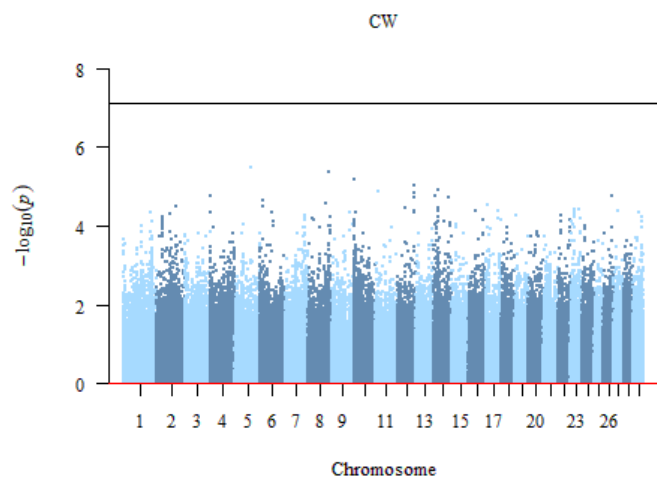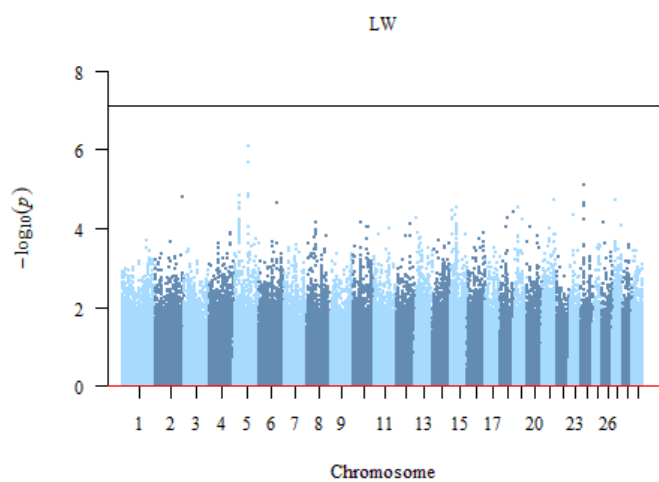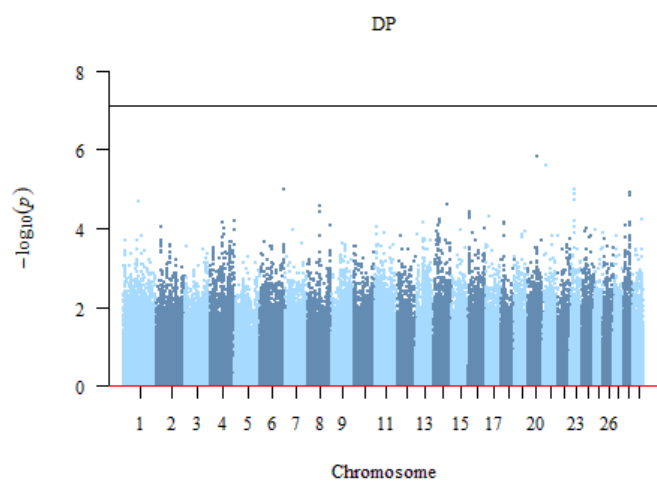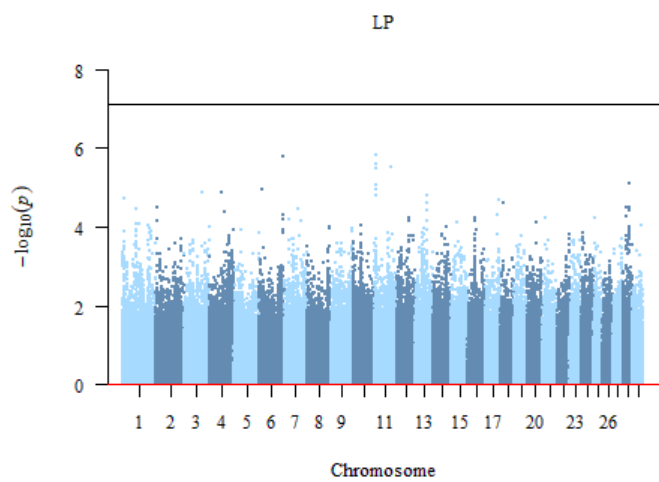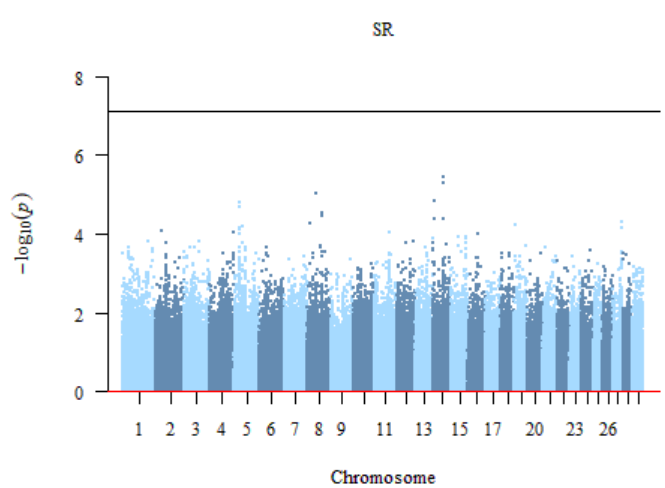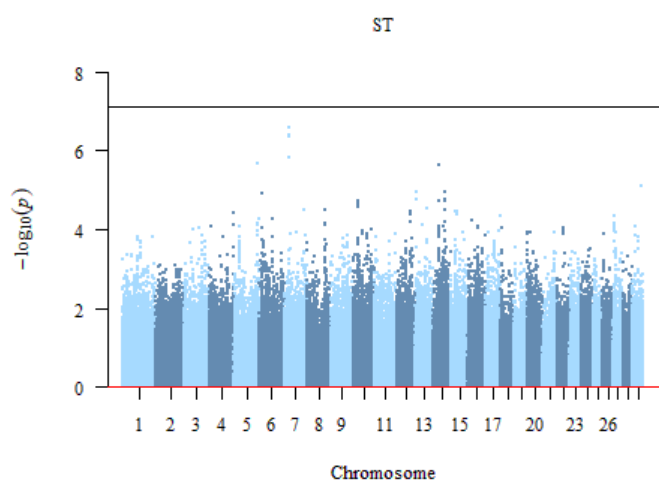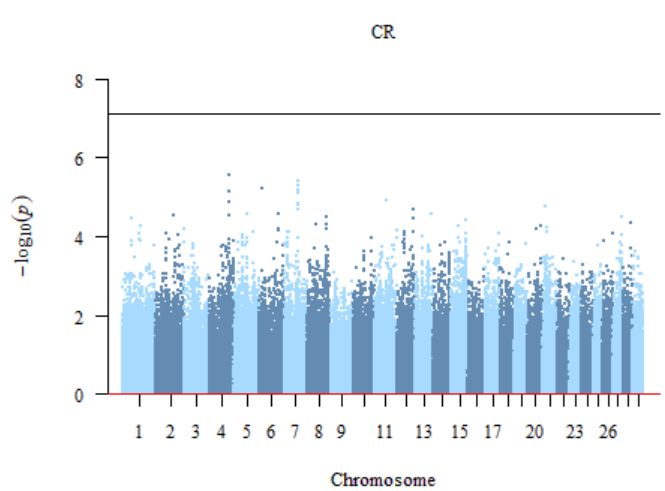

TD

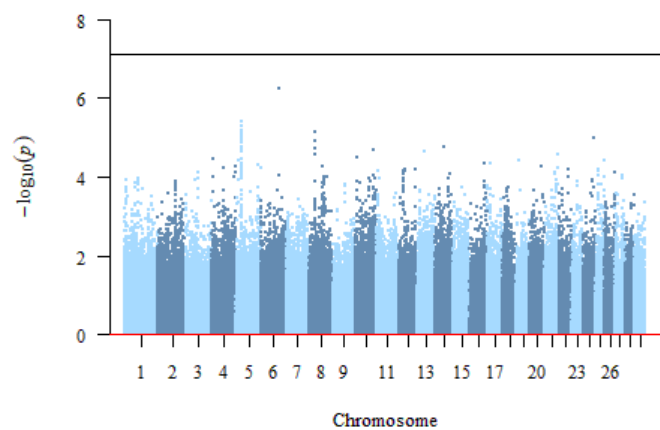

EMA12

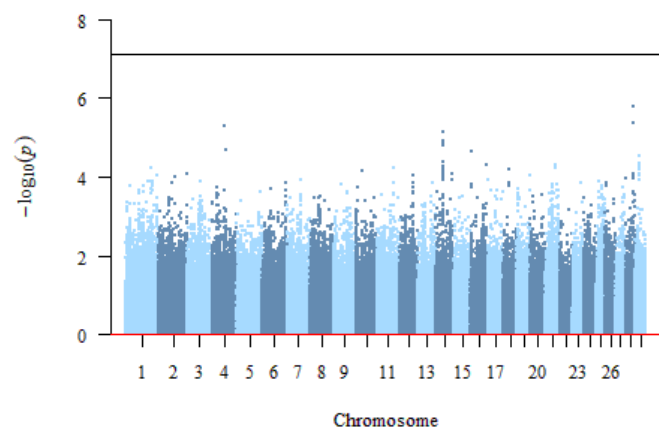

RMW

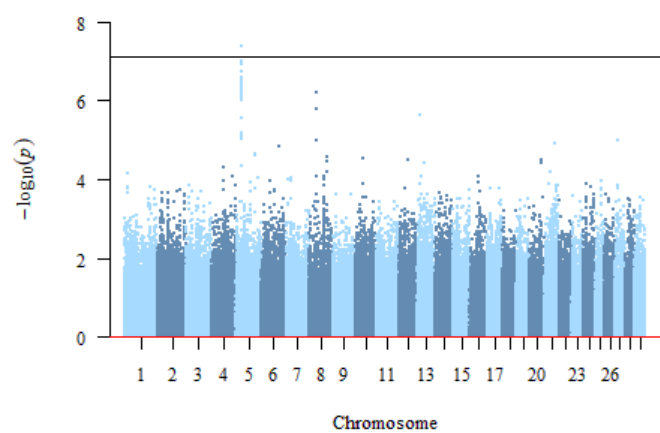

EMA3

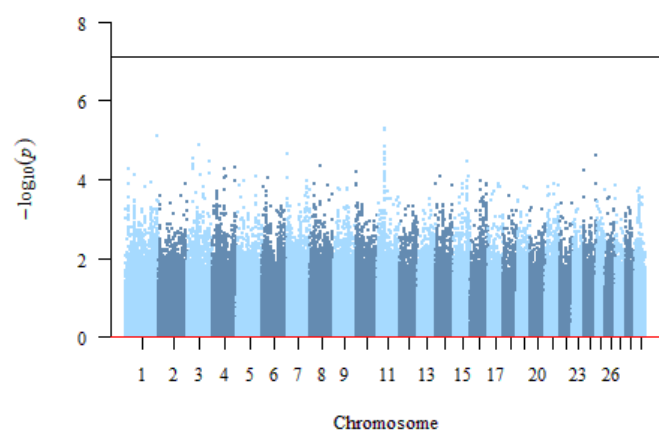

MB

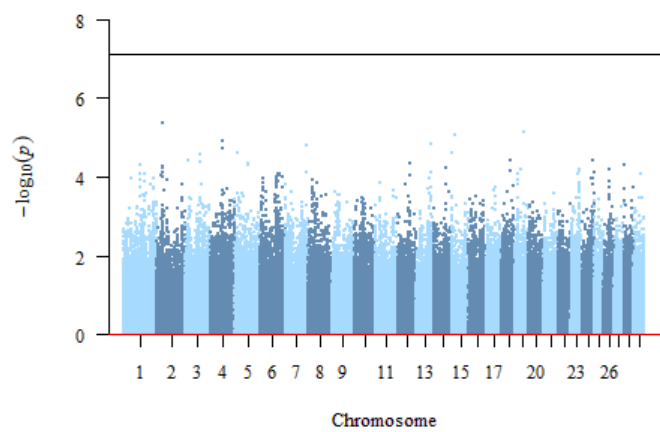

Supplement: Supplementary file 1 [file animals-11-01890-s001.zip › Figure S2.pdf]

**a**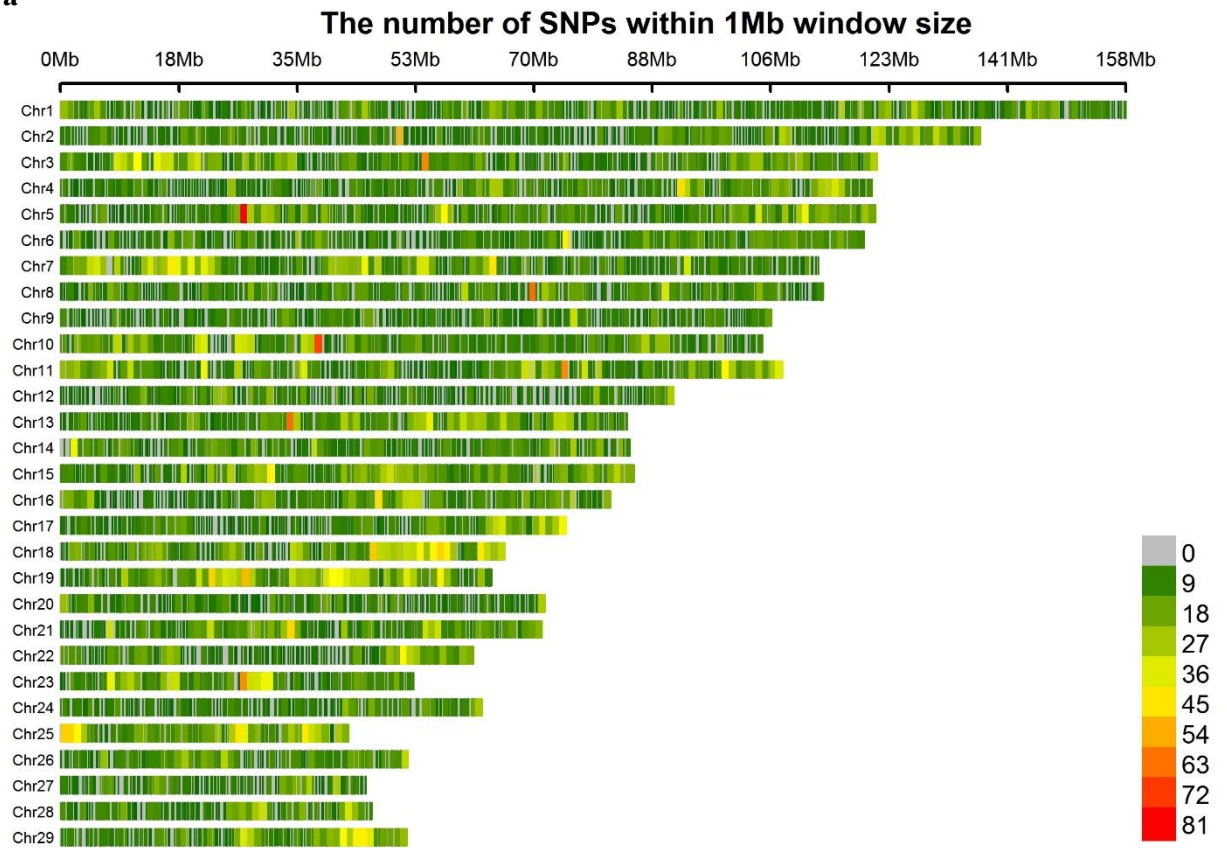**b**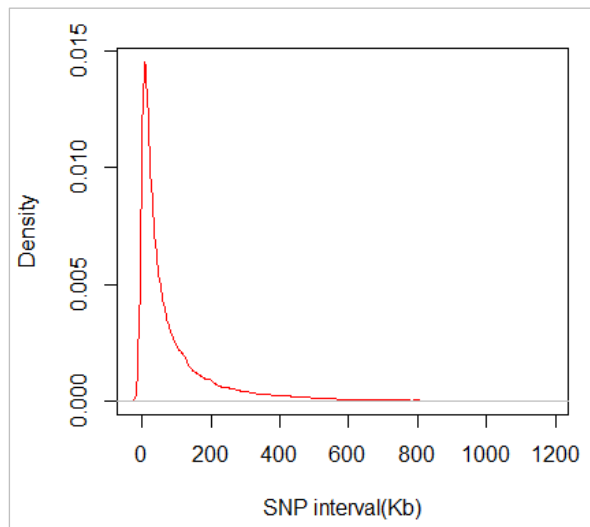**c**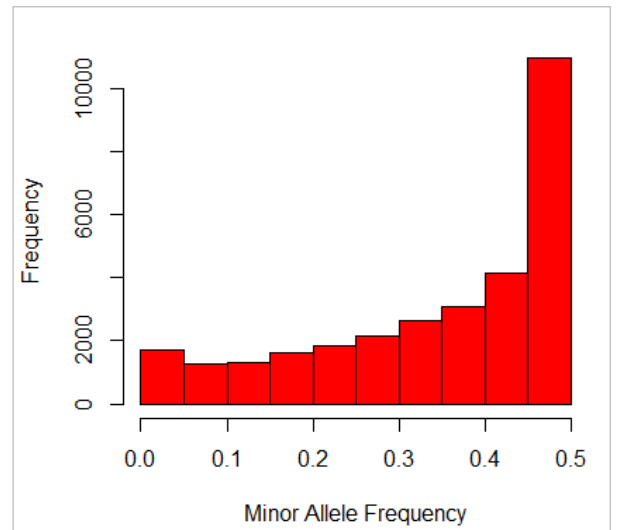

Supplement: Supplementary file 1 [file animals-11-01890-s001.zip › Figure S3.pdf]
